# Structural characterization of semi-heusler/light metal composites prepared by spark plasma sintering

**DOI:** 10.1038/s41598-018-29479-3

**Published:** 2018-07-24

**Authors:** Jaromír Kopeček, Kristína Bartha, Radek Mušálek, Zdeněk Pala, Tomáš Chráska, Přemysl Beran, Vasyl Ryukhtin, Pavel Strunz, Jaroslava Nováková, Josef Stráský, Pavel Novák, Oleg Heczko, Michal Landa, Hanuš Seiner, Miloš Janeček

**Affiliations:** 1 Institute of Physics, The Czech Academy of Sciences, Na Slovance 1999/2, Prague, 182 00 Czech Republic; 2 Department of Physics of Materials, Charles University, Ke Karlovu 5, Prague, 121 16 Czech Republic; 3 Institute of Plasma Physics, The Czech Academy of Sciences, Za Slovankou 1782/3, Prague, 182 00 Czech Republic; 4 Nuclear Physics Institute, The Czech Academy of Sciences, Řež 130, Řež, Prague, 250 68 Czech Republic; 5 Department of Surface and Plasma Science, Charles University, V Holešovičkách 2, Prague, 180 00 Czech Republic; 6 Department of Metals and Corrosion Engineering, University of Chemistry and Technology Prague, Technická 5, Prague, 166 28 Czech Republic; 7 Institute of Thermomechanics, The Czech Academy of Sciences, Dolejškova 1402/5, Prague, 182 00 Czech Republic

## Abstract

A composite of powders of semi-Heusler ferromagnetic shape memory and pure titanium was successfully prepared by spark plasma sintering at the temperature of 950 °C. Sintering resulted in the formation of small precipitates and intermetallic phases at the heterogeneous interfaces. Various complementary experimental methods were used to fully characterize the microstructure. Imaging methods including transmission and scanning electron microscopy with energy dispersive X-ray spectroscopy revealed a position and chemical composition of individual intermetallic phases and precipitates. The crystalline structure of the phases was examined by a joint refinement of X-ray and neutron diffraction patterns. It was found that Co_38_Ni_33_Al_29_ decomposes into the B2-(Co,Ni)Al matrix and A1-(Co,Ni,Al) particles during sintering, while Al, Co and Ni diffuse into Ti forming an eutectic two phase structure with C9-Ti_2_(Co,Ni) precipitates. Complicated interface intermetallic structure containing C9-Ti_2_(Co,Ni), B2-(Co,Ni)Ti and L2_1_-(Co,Ni)(Al,Ti) was completely revealed. In addition, C9-Ti_2_(Co,Ni) and A1-(Co,Ni,Al) precipitates were investigated by an advanced method of small angle neutron scattering. This study proves that powder metallurgy followed by spark plasma sintering is an appropriate technique to prepare bulk composites from very dissimilar materials.

## Introduction

Advanced composite materials, when properly designed, offer unique macro-scale behaviours different from the micro-scale properties of the individual constituents. This opens a wide range of possibilities for development of new materials with novel functionalities. On the other hand, the effort to combine materials with very dissimilar physical and mechanical properties into a compact composite structure may encounter considerable technical issues, especially when the characteristic length in the composite is required to appear at the scale of micrometers or even nanometers. Nevertheless, both Nature and advanced technologies are capable of producing such materials.

One of the novel and recently quickly expanding techniques of the production of bulk microcrystalline composite materials is their compaction from powders using a method of spark-plasma sintering (SPS)^1^. The SPS technique, also called pulsed electric current sintering (PECS) or field assisted sintering technique (FAST), is a relatively novel technique of powder consolidation. During SPS, applied pulsed or continual direct current passes directly through a conductive material and heats it up internally by the Joule heat^1–3^. Physical processes occurring during SPS are still not fully understood. However, the diffusion leading to material compaction is by several orders of magnitude faster during SPS than in other compaction techniques. As a result, the material can be sintered in much shorter times and under lower temperatures than by any other compaction method^1,3,4^. SPS technique can be used to produce compact samples of unusual materials combination^5,6^. For example, it is possible to prepare functionally graded Cu/W composite combining machinability of copper with superior hardness and temperature resistance of tungsten^7^ or a mixture of Ni-based and Fe-based glassy alloy powders to prepare a dual-phase composite with unique high-strength properties^8^. Apart from mechanical or thermal properties of composites, more peculiar physical properties can find their use in engineering materials – this might include for instance ferromagnetism, thermoelectric properties or shape memory effect. SPS has been already used for manufacturing of ferroelectric composites^9^, magnetoelectric ceramic composites^10^ or soft magnetic composite materials^11,12^. The possibilities of mixing a material with a distinct functional property (ferromagnetism, shape memory) with a light-weight metal remain almost unexplored. Basic characterization of Mg matrix reinforced by NiMnGa was presented in^13^. A more elaborated example of this approach is presented in a pioneering work of Mizuuchi *et al*.^14^ in that a mixture of shape memory alloy and light-weight magnesium alloy AZ31 was successfully manufactured. Finally, an attempt to maintain ferromagnetic properties of Ni-Cu-Zn ferrite in Mg matrix is reported in^15^. All three mentioned studies report an evolution of intermediate interface phases between the constituents, while a detailed analysis of these layers is excessively complicated. The effect of spark plasma sintering on microstructure, phase composition and thermoelectric properties of a half-Heusler compound were investigated in few previous studies^16–18^, but these researches did not exploit the ability of SPS for sintering materials mixtures. Such approach was used in a unique study which reported that thermoelectric properties of a half-Heusler compound may be improved by sintering with a full-Heusler phase using SPS^19^.

It should be pointed out, that SPS plays an important role in the process by being a part of the synthesis of the material rather than solely a densification technique^19^. In the case of sintering of very dissimilar materials, the interface phases, which affect resulting physical properties, are likely to occur. As a consequence, it can be extremely difficult and complicated to characterize properly and completely these interface layers.

Recently, Koller *et al*.^20^ explored the possibility of combining a light metal with a ferromagnetic semi-Heusler alloy, in order to obtain a light-weight metal composite with locally ferromagnetic behaviour. In such composite, the structural damage could be identified by Barkhausen noise detection or it might be usable for light-weight magnetic shielding applications. Furthermore, considering that CoNiAl is a ferromagnetic material and titanium is well known as a biocompatible metal, the composite made of these two material may find its use in novel medical applications, such as cancer treatment by so-called magnetic hyperthermia^21,22^. The authors reported on mechanical properties and selected functional properties of this composite, particularly ferromagnetism and ferroelasticity, noticing the existence of non-magnetic layers at the semi-Heusler/light metal interface^20^. In this study, we present a detailed analysis of the crystal structure and chemical composition of the same composite, i.e. the Co_38_Ni_33_Al_29_-Ti SPS processed composite, hereafter referred to as CoNiAl-Ti only.

As the semi-Heusler component, the off-stoichiometric ferromagnetic shape memory alloy Co_38_Ni_33_Al_29_ was chosen, which was studied in^23^ and in our previous works^24,25^. Despite this class of alloys was rarely prepared by powder metallurgy^26,27^, PM produced Co-Cr-Al based oxide dispersion strengthened alloys were investigated for applications in medicine^28^ and even cobalt superalloys containing titanium were studied^29^. Titanium was chosen as a light-weight filler instead of aluminium or magnesium to preserve high-temperature properties of the composite. The commercially pure α-titanium (CP-Ti) Grade 2 and Ti-based alloys were proved to be consolable by SPS^30–34^.

Co_38_Ni_33_Al_29_ alloy undergoes a cubic-to-tetragonal transition at low temperatures^23^, which is a shape memory effect performing transformation. However, this transition can be observed only in Co-Ni-Al alloy annealed at temperatures above approx. 1000 °C, while the as-cast material used in this study is stable in the cubic B2 phase down to 10 K^25^. Phase composition of Co-Ni-Al system is rather complicated. Kainuma *et al*. already reported in^35^ (except of B2 cubic or L1_0_ tetragonal matrix) two types of non-transforming precipitates: disordered FCC cobalt solid solution particles (so called disordered A1) and L1_2_ ordered (Co,Ni)_3_Al particles. The disordered A1 particles appear in slowly solidified samples, whereas the ordered L1_2_ precipitates were observed rather in annealed samples. This observation is consistent with our previous measurements^24^. The level of ordering is below the detection limit of the X-ray diffraction technique^36–38^, whereas transmission electron microscopy was able to find different ordering of atoms in particles of different morphology^24^.

The phase diagram of Al-Co-Ni system was reported in^39^. It contains wide strips of single phase areas, where the lattice parameters change with changing ratio of fully miscible cobalt and nickel atoms. The quaternary phase diagram of Al-Co-Ni-Ti keeps the symmetry given by cobalt and nickel miscibility and by competition of aluminium and titanium elements during the creation of ordered phases. Despite the cobalt-nickel miscibility, the symmetry of calculated phase diagrams suggests the cobalt and nickel segregation into phases B2/L2_1_ (NiAl – Ni_2_AlTi – Co_2_AlTi – CoAl)^40^ or into phases A1(FCC)/L1_2_ ((Co,Ni)_3_(Al,Ti)^41^. The segregation in ternary Al-Ni-Ti based systems was described in^42^, predominantly as the separation of aluminium and titanium elements. The latest review of quaternary diagram of Al-Co-Ni-Ti can be found elsewhere^43^. The mass density of Co_38_Ni_33_Al_29_ alloy (*ρ* = *6*.*95 gcm*^*−3*^) is significantly lower than that of single-element ferromagnetic metals (Ni, Fe, Co) due to the light-weight aluminium, and mechanical properties of the selected Co_38_Ni_33_Al_29_ alloy are favourable up to high temperatures.

The aim of the present study is to determine the crystalline structure and chemical composition of constituent powders and their interfaces in detail in order to understand the processes during sintering. A wide palette of complementary experimentally advanced techniques was applied for a detail structural characterization of such complicated interfacial structure. In particular, X-ray diffraction (XRD) and neutron diffraction (ND) are appropriate techniques to determine phase composition and overall volume fraction of phases, but are unable to localize respective phases. On the other hand, scanning electron microscopy (SEM) is capable to depict individual interface layers, but fundamentally cannot identify phases. Only SEM analytical methods of electron backscatter diffraction (EBSD) and energy dispersive spectroscopy (EDS) can identify and localize individual phases and determine their chemical composition, respectively. Transmission electron microscopy (TEM) technique is able to confirm positions of identified phases and to determine their lattice parameters from the electron diffraction patterns. Finally, small angle neutron scattering (SANS) method is able to characterize the structure properties of small particles and precipitates in the material.

## Results and Data Evaluation

### Microstructure

**Figure 1:**
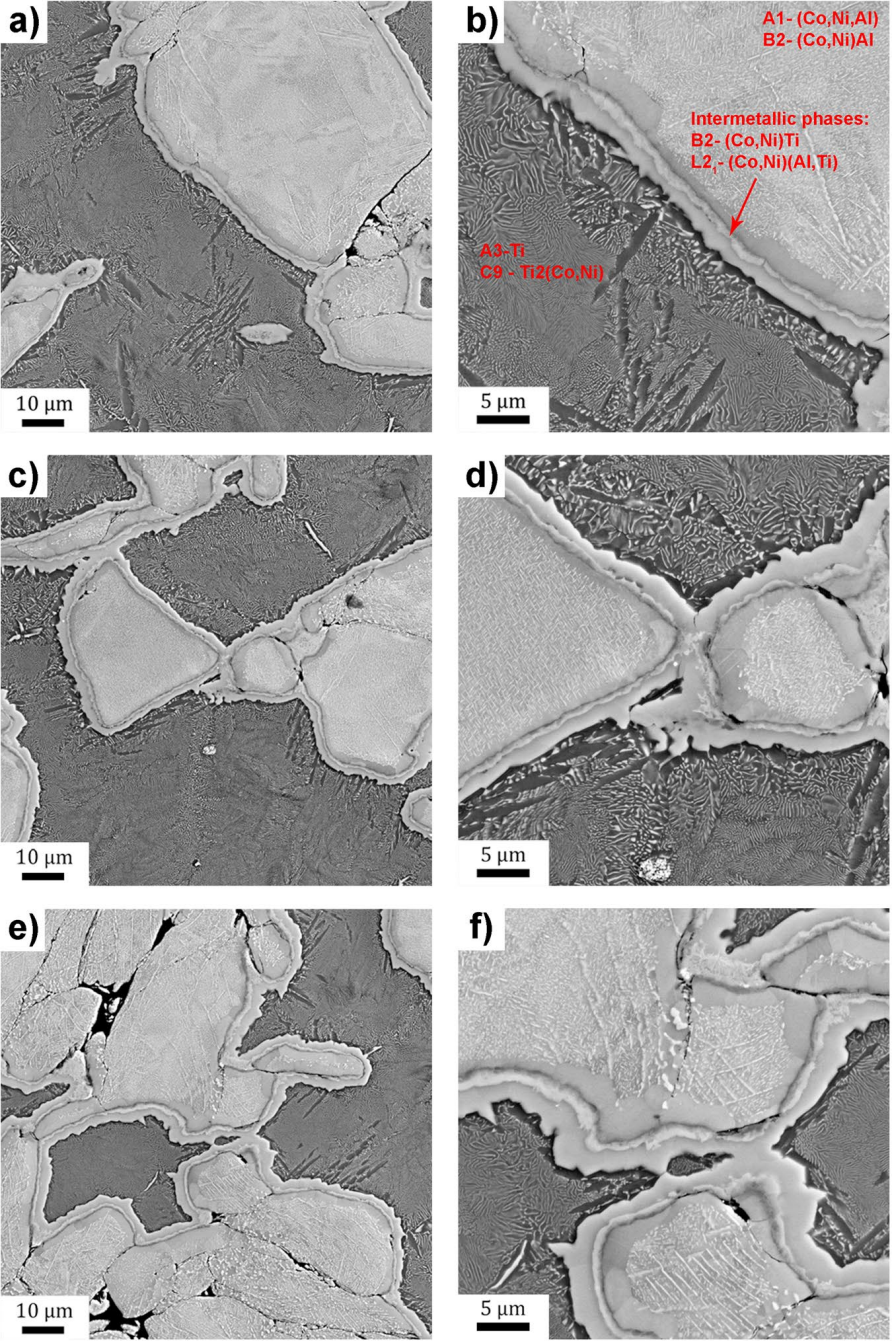
SEM observation of the CoNiAl:Ti composite. (**a**) CoNiAl:Ti 1:2, sintered for 1 min, overview, (**b**) CoNiAl:Ti 1:2, sintered for 1 min, detail, (**c**) CoNiAl:Ti 1:1, sintered for 1 min, overview, (**d**) CoNiAl:Ti 1:1, sintered for 1 min, detail, (**e**) CoNiAl:Ti 2:1, sintered for 1 min, overview, (**f**) CoNiAl:Ti 2:1, sintered for 1 min, detail.

SEM micrographs of all three materials (CoNiAl:Ti-1:2, 1:1 and 2:1) sintered for 1 minute at the temperature of 950 °C by SPS processing are shown in Fig. 1a–f. In Fig. 1b, the overview of present phases is provided. Due to chemical contrast CoNiAl particles appear bright, while Ti ones are darker. All samples are compact and dense without apparent voids indicating successful densification process. Some small voids are seen between CoNiAl particles, because the mixing energy as driving force for coalescence is negligible. On the other hand, the temperature of 950 °C is sufficiently high enough to allow full sintering of titanium. It exceeds both the β-transus temperature in pure Ti (885 °C) and the eutectic temperatures in titanium rich part of Ti-Co (685 °C) and Ti-Ni (765°) systems.

White dots appeared inside titanium grains which were not present in the initial powder. According the phase diagram these white dots might be Ti_2_Co or Ti_2_Ni intermetallic phases. Similarly looking dots within CoNiAl grains are, however, of a different origin. According to the phase diagram described in^36,37^, these were identified as FCC cobalt solid solution (A1).

The interfaces between CoNiAl particles and Ti can be identified as several layers of various intermetallic phases, which are characterized in detail in the following sections. Unfortunately, the dependence of the thickness of these interface layers on sintering time cannot be unambiguously determined from the SEM imaging. On the other hand, indirect magnetization measurements indicate a reduced magnetization for samples sintered for longer times^20^. This suggests that the volume fraction (and therefore also the thickness) of non-magnetic interface layers increases with increasing sintering time.

### X-ray and neutron diffraction measurements

X-ray diffraction and neutron diffraction measurements were employed to determine phase composition and volume fractions of individual phases of all prepared samples.

**Figure 2:**
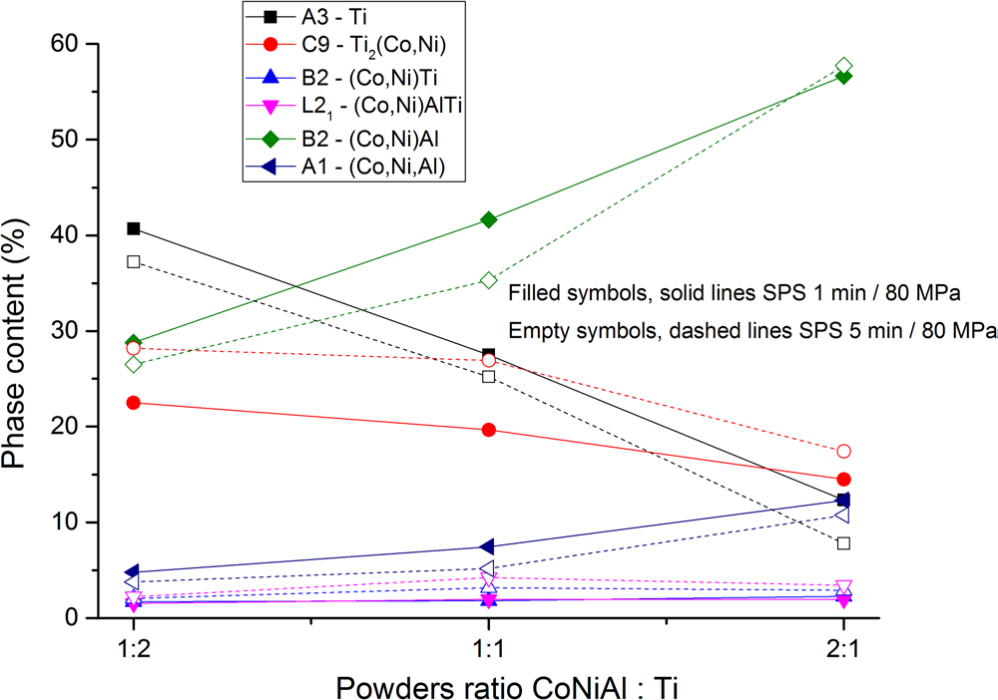
Phase content and its evolution for various powder ratios and sintering times obtained from combined XRD and NRD.

The evolution of the volume fraction of individual phases as a function of CoNiAl:Ti ratio from joined refinement of both XRD and ND measurements is shown in Fig. 2. The phases whose volume fraction increases with CoNiAl:Ti ratio content, were formed in CoNiAl grains and vice versa. The matrix of CoNiAl grains was identified as B2-(Co,Ni)Al phase. Its volume fraction increases from 30% for 1:2 CoNiAl:Ti ratio to 60% for 2:1 ratio and slightly decreases with increasing sintering time. The amount of particles in large CoNiAl grains (white dots in Fig. 1) identified as FCC cobalt solid solution (A1) was 3% and 10% for the ratio of CoNiAl:Ti 1:2 and 2:1, respectively. The fraction of this A1 phase decreases with increasing sintering time.

The phase of titanium is A3 hexagonal (α-Ti) and its volume fraction decreases with increasing sintering time and with decreasing relative content of Ti powder from 40% to 10%. The most pronounced precipitation of the intermetallic phases can be observed inside the titanium grains and in layers on their surfaces. The precipitation product can be easily identified as the C9-Ti_2_(Co,Ni) phase, because of its high lattice parameter (approx. 11.3 Å) and a space group Fd-3m. Its amount is the highest for 1:2 ratio −25%, and decreases to 15% for 2:1 powders ratio. The amount of this phase increases also with increasing sintering time. The phase identification is consistent with the SEM observation, cf. Figure 1.

Identification of intermetallic phases consisting of titanium and any of Co, Ni and Al elements growing in the contact or interfacial regions is more complex. Interfacial phases were finally identified as (from Ti side): C9-Ti_2_(Co,Ni); B2-(Co,Ni)Ti and L2_1_-(Co,Ni)(Al,Ti). The complete identification of these phases requires combined experimental techniques of XRD, ND, EDS and TEM as described below.

The L2_1_-(Co,Ni)(Al,Ti) phase was determined by EDS, as it contains lower amount of aluminium compared to B2-(Co-Ni)Al and significant amount of titanium. The existence of this phase is consistent with the phase diagram described in^40^. The L2_1_ phase has similar structure to B2 phase, but complete ordering is not achieved, arguably due to short time of sintering.

### Correlated EBSD/EDS measurements and SEM observation

**Figure 3:**
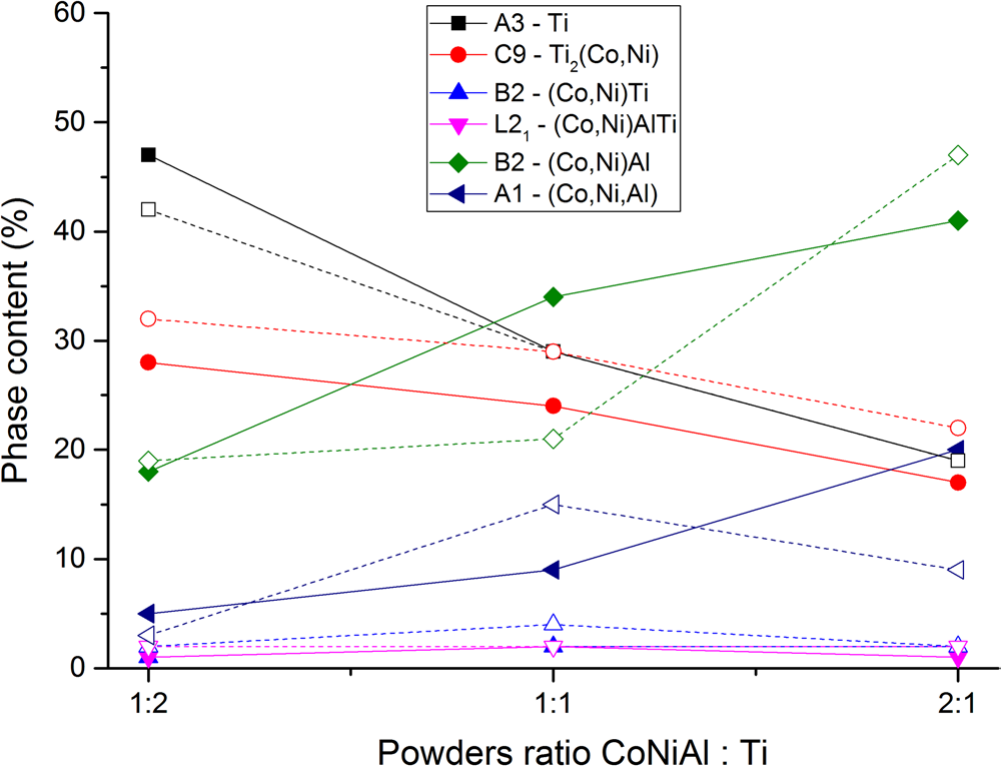
The phase content and its evolution for various powder ratios and sintering times obtained from combined EBSD/EDS measurement.

The evolution of the volume fraction of individual phases as a function of CoNiAl:Ti ratio was also determined from combined EBSD/EDS measurement. The results are summarized in Fig. 3. The comparison of Figs 2 and 3 indicates that the results of XRD/ND and EBSD/EDS are consistent.

Correlated EBSD/EDS mapping was performed for all samples to support the diffraction experiments with the direct microscopic observations. The phases were identified using “Chemical Indexing Scan” (so-called ChI-scan), i.e. using the correlation of crystalline structure determined by EBSD with chemical composition determined by EDS.

**Figure 4:**
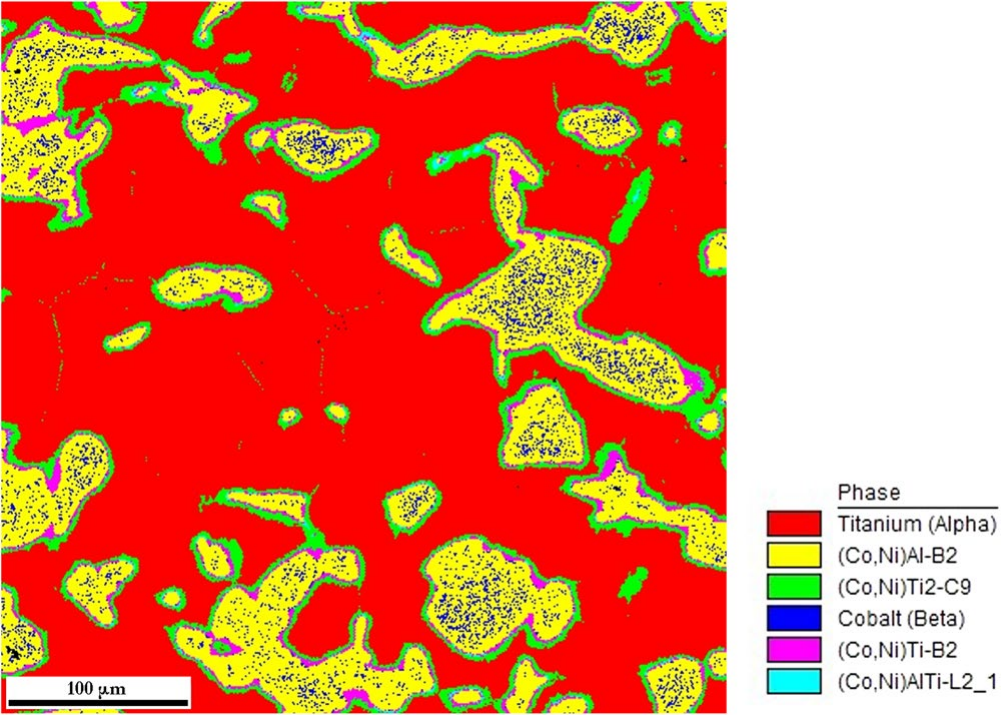
EDS map from combined EBSD/EDS measurement of sample CoNiAl:Ti = 1:2, SPS sintered for 5 minutes (overview). The corresponding colour code is displayed on the right side of phase map.

**Figure 5:**
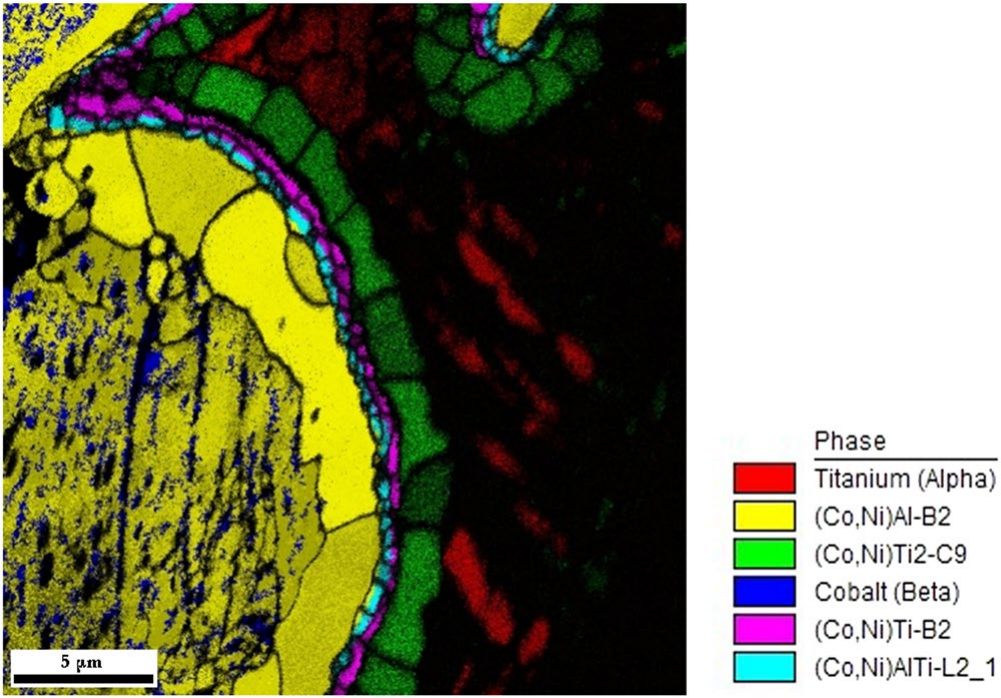
EDS map from combined EBSD/EDS measurement of sample CoNiAl:Ti = 1:2, sintered for 5 minutes (detail). The corresponding colour code is displayed on the right side of phase map. Coloured image is overlaid by greyscale map of confidence index – the parameter which limits the reliability of the indexation.

Two examples of the experiment are shown in Fig. 4 (overview) and in Fig. 5 (detail). In Fig. 4, the distribution of identified phases is shown in a colour map. In Fig. 5, the colour map is overlaid by a greyscale map corresponding to the “confidence index” of the EBSD indexation. It can be observed that especially the titanium phase is poorly diffracting, which often prevents the determination of the crystal lattice orientation of this phase. One reason is the metallographic preparation of the sample – the titanium phase is comparatively soft and tough when compared to all other present phases and therefore polishing of Ti particles is not perfect. Another reason for poorly diffracting Ti phase is the presence of tiny C9-Ti_2_(Co,Ni) particles in A3-Ti matrix. The interaction volume of EDS technique is bigger than the typical size of C9-Ti_2_(Co,Ni) particles and therefore these particles cannot be identified and the identification of matrix phase is blurred. Similarly, due to large interaction volume of EDS method, the identification of A1-(Co,Ni,Al) particles within B2-(Co,Ni)Al matrix is also problematic. On the other hand, the determination of interfacial phases employing the combined EBSD/EDS experiment was quite successful and consistent with the XRD/ND joint refinement results (cf. Fig. 2). The measurement also showed that the interface layers are polycrystalline and individual grains can be recognized by EBSD measurement.

As suggested in Fig. 4 and revealed in Fig. 5 in detail, larger Ti_2_(Co,Ni) particles in the Ti matrix cluster in chains along grain/particle boundaries of Ti. EDS mapping of nickel (not shown) proved the higher concentration of Ni along grain boundaries (GBs) in Ti, suggesting a long-range diffusion of Ni along GBs in Ti. On the other hand, Co content along grain boundaries was not increased and GB C9-Ti_2_(Co,Ni) phase contains more Ni at the expense of Co. A different situation occurs for the two other intermetallic phases (B2 and L2_1_) visible as thin ribbons on the interface between CoNiAl and Ti grains. These phases seem to contain Co and Ni equally.

### Detailed chemical composition analysis of the interface

**Figure 6:**
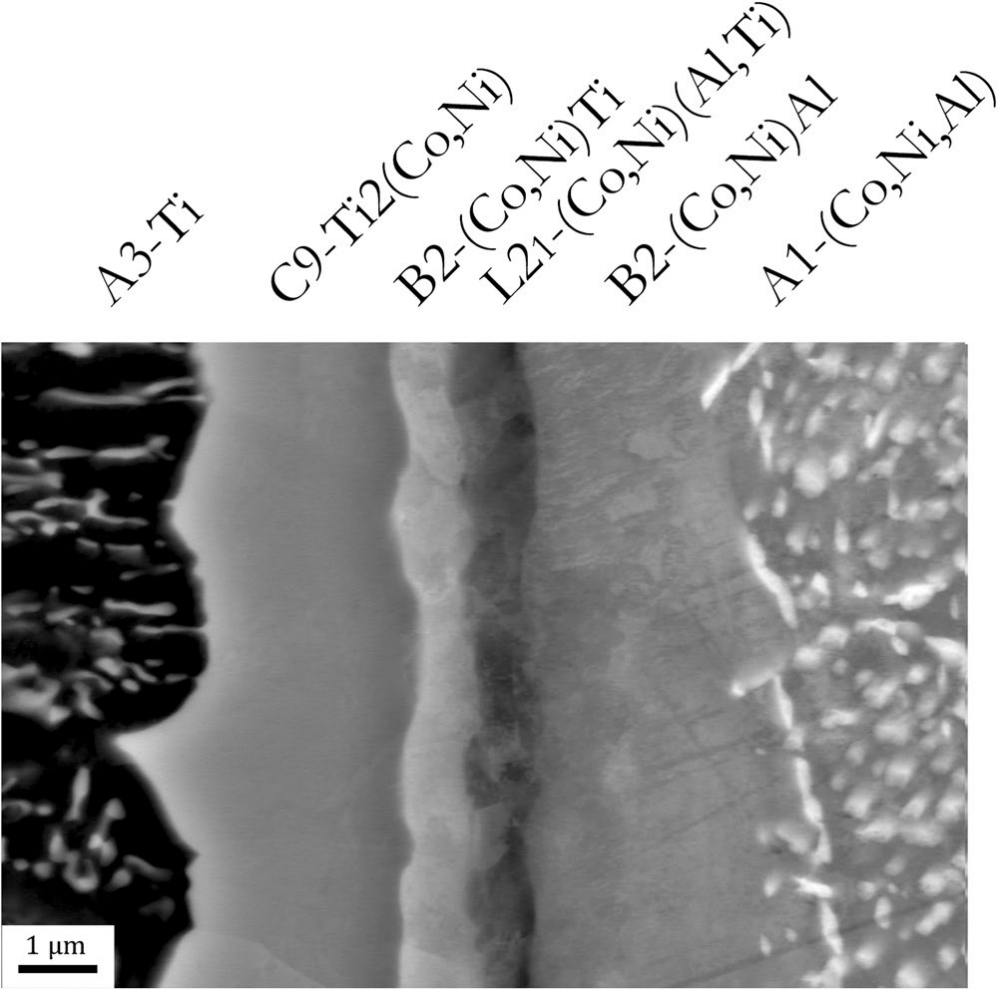
Detailed SEM micrograph of inter-powder boundary identifying all observed phases (cf. Fig. 8 below – STEM micrograph for comparison).

Figure 6 shows the interface layers between a Ti particle (left) and a CoNiAl particle (right) employing SEM with a high magnification and resolution. The interface layers can be easily recognized. Based on the diffraction and EBSD/EDS experiments, the layers can be associated with the identified phases. However, the thickness of each layer cannot be properly determined from the image, because it is not assured that the layers are cut perpendicularly.

**Figure 7:**
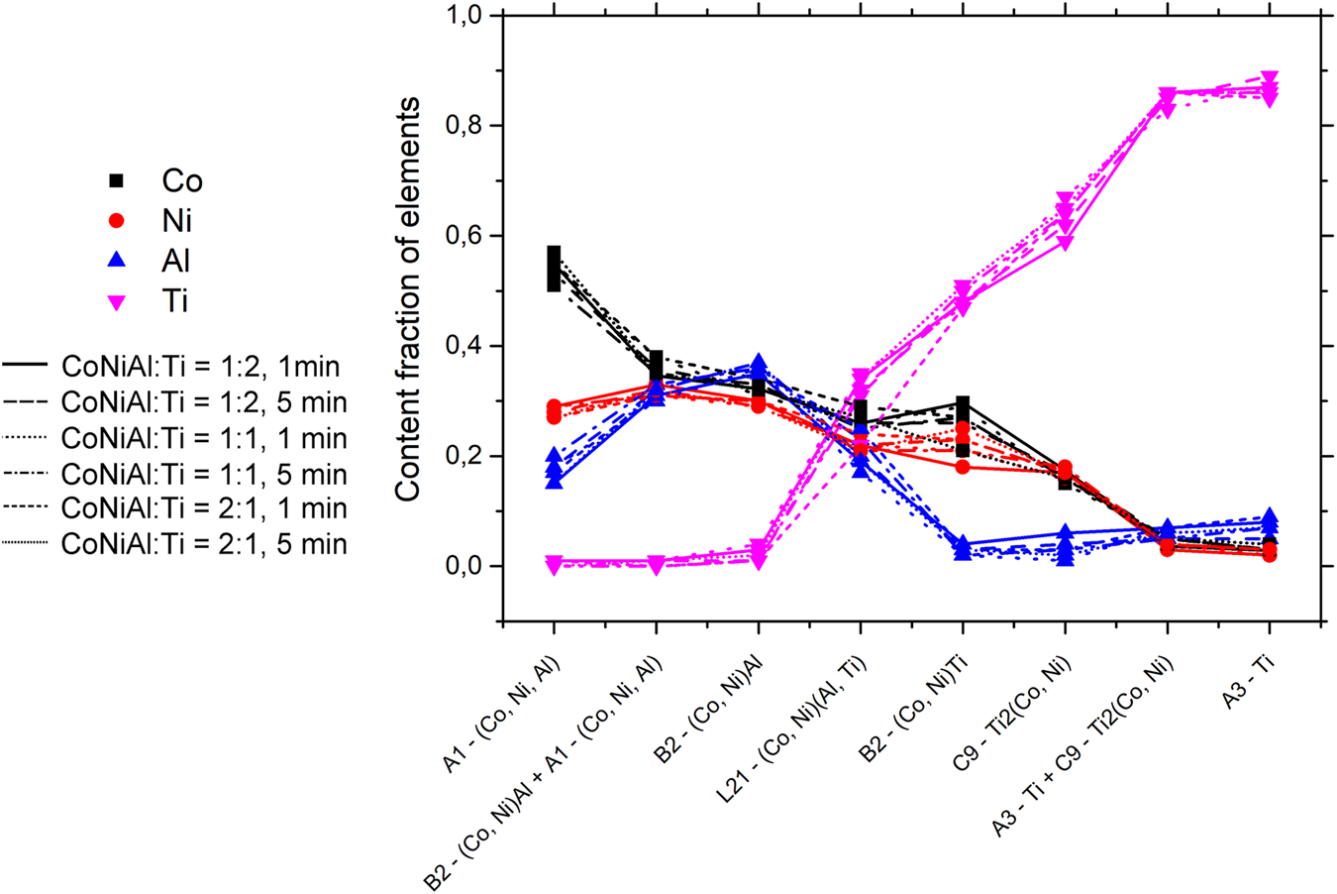
Chemical composition of individual identified phases determined by EDS point analysis (several analyses per phase were performed in each sample). All prepared samples were analysed.

EDS method was used again, in this case to determine the chemical composition of the individual interface layers. Despite it was technically possible to measure the line scan by EDS, the results would be subjected to large error since the size of the layers are comparable to the interaction volume. Instead, several point scans were measured for each phase and each condition. The results of this experiment are summarized in Fig. 7. The points in the graph correspond to the average concentration of each element in each phase for each prepared sample (SPS condition). The lines between points serve as a guide for the eye only.

The composition of A1-(Co,Ni,Al) phase was determined from the biggest particles shown as white particles in Fig. 1f, while the composition of A3-Ti phase was determined from C9-Ti_2_(Co,Ni)-free areas (bigger black areas shown in Fig. 1b). The chemical composition of interfacial layers seems to be independent of CoNiAl:Ti ratio and also of sintering time.

### Confirmation of phase composition by TEM

**Figure 8:**
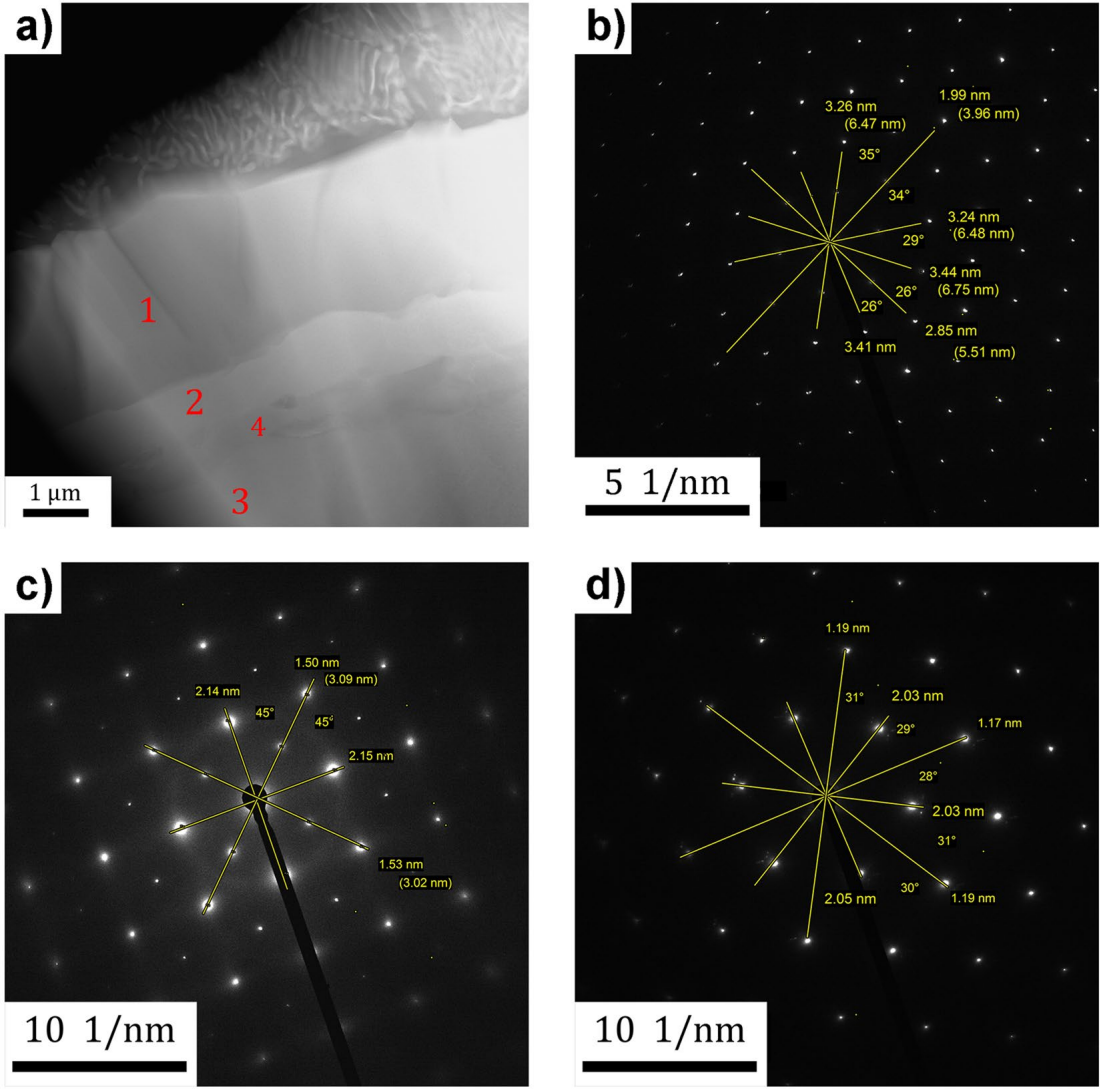
TEM observation of the sample CoNiAl:Ti 2:1, 5 min. (**a**) Detailed STEM micrograph of sample CoNiAl:Ti 2:1, 5 min, showing Ti and intermetallic part; numbers are representing the layers from that the SAED patterns were taken (**b**–**d**), (**b**) 1-[011] zone axis SAED pattern of the phase C9 – Ti_2_(Co,Ni), (**c**) 2-[001] zone axis SAED pattern of the phase B2 – (Co,Ni)Ti, (d) 3-[111] zone axis SAED pattern of the phase B2 – (Co, Ni)Al.

An additional TEM study was performed to confirm the phase identification of interface layers by SEM. The STEM micrograph (Fig. 8a) shows the individual interfacial layers from the Ti side (upper part of image) to the CoNiAl side. Each interfacial layer was analysed by selected area electron diffraction (SAED). Examples of SAED patterns are shown in Fig. 8b–d. Electron diffraction confirmed the hexagonal A3-Ti phase in titanium grains (not shown here) and adjacent intermetallics C9-Ti_2_(Co,Ni) (Fig. 8b). The matrix of CoNiAl grains and the phase B2-(Co,Ni)Al were identified as well (Fig. 8d). The most valuable output of the TEM study was the identification of the central layer between both types of materials (appearing brighter in Fig. 6) to be B2-(Co,Ni)Ti with the space group Pm-3m in agreement with Fig. 5. The [001] zone axis SAED pattern displayed in Fig. 8c corresponds to B2-(Co,Ni)Ti phase with lattice parameter 3,01 Å. However, the structure of neighbouring phase L2_1_ (marked with number 4 in Fig. 8a) could not be identified by TEM due to its fragmented and deformed microstructure. The structures of B2 and L2_1_ phases are very similar, i.e. cubic differing only by their chemical ordering and space groups. Moreover, the lattice parameters of these two phases are also comparable. Previous experiments by SEM and EDS proved only that such a layer exists and differs chemically from the neighbouring B2 phases.

### Investigation of precipitates by small angle neutron scattering

**Figure 9:**
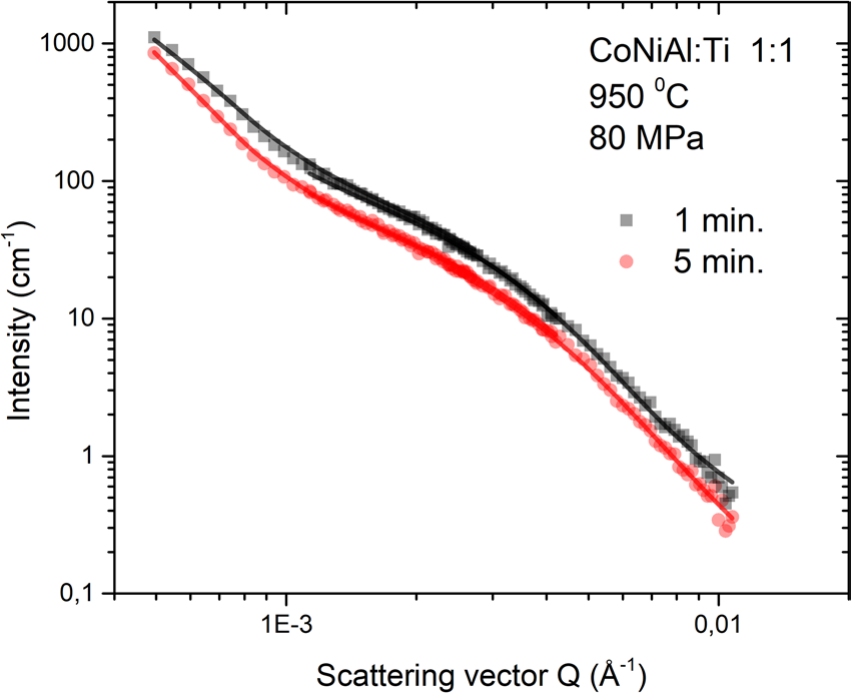
Measured (symbols) and fitted (lines) scattering curves from CoNiAl:Ti sample 1:1 for sintering time of 1 and 5 min.

Small angle neutron scattering (SANS) experiment was used to reveal the dependence of the structure of small precipitates on the CoNiAl:Ti ratio and the sintering time. As an example of the measured and fitted SANS data, scattering curves from CoNiAl:Ti sample 1:1 and sintering times of 1 and 5 min are shown in Fig. 9. Similar results were also obtained for ratios 1:2 and 2:1 (not shown here). The scattering in the low-*Q* region (up to 1 × 10^−3^ Å^−1^) is caused by large particles (exceeding 1 μm in size) and pores. The intensity is roughly proportional to their specific interfaces in the surrounding matrix. The scattering of the diffraction vector *Q* in the range of 1 × 10^−3^–1 × 10^−2^ Å^−1^ is caused by small particles of the size of tens up to couple of hundreds nanometres. From the plot in the Fig. 9 it can be inferred that the overall intensity of scattering in both Q-regions decreased significantly in specimen sintered longer time (5 min) as compared to the specimen sintered shorter time (1 min). The decrease of the intensity in the low-*Q* region is caused by (i) coarsening of the large precipitates and (ii) decrease of the amount of pores formed during sintering. The decrease of the intensity in the *Q*-range of 1 × 10^−3^–1 × 10^−2^ Å^−1^ is caused by the decrease of the volume fraction of small precipitates, i.e. either C9-Ti_2_(Co,Ni) or A1-(Co,Ni,Al) precipitates. The size of these particles was not significantly changed with the sintering time as the shape of the scattering curve remains unchanged.

Scattering contrast of the particles of individual phases in the surrounding matrix must be considered to interpret the measured SANS data quantitatively. The involved scattering particles could be either C9-Ti_2_(Co,Ni) in A3-Ti grains, or A1-(Co,Ni,Al) in B2-(Co,Ni)Al matrix, or both (cf. Fig. 1). When EDS results (see Fig. 7) are employed for scattering length density (SLD) calculation of the individual phases, the obtained SLD difference is 1.9 × 10^−6^ Å^−2^ and 0.9 × 10^−6^ Å^−2^ for C9 precipitates with respect to the Ti matrix and for A1 precipitates in CoNiAl grains, respectively. Since the scattering intensity is proportional to the square of SLD difference, the dominant contribution to the scattering is clearly from the C9 precipitates. Therefore the SANS data were evaluated by fitting of a single population of particles with the shape of ellipsoids with fixed large axis size and free small axis size.

The results of the SANS measurements indicate that the mean size of precipitates does not change significantly with the sintering time, and remains in the range of 105–150 nm in all cases.

The volume fraction of C9 precipitates decreases with increasing sintering time from approximately 15% to 10%. This seems to be in contradiction with the results of neutron diffraction measurements which indicate the opposite trend. However, neutron diffraction determines the total volume fraction of C9 phase which consists of a massive layer at CoNiAl-Ti interface and of fine C9 precipitates in grain interior, see Fig. 5. On the contrary, SANS is sensitive solely to C9 precipitates. As a consequence, the thickness of C9 phase layer on the CoNiAl-Ti interface is expected to increase with increasing sintering time^20^, while fine C9 precipitates near the interfacial layer between Ti and CoNiAl grains tend to disappear.

## Discussion

The ability of SPS to produce compact samples from very dissimilar materials is very useful albeit it grossly complicates the structural analysis of manufactured composites. On one hand, SPS leads to efficient compaction of materials, while on the other hand, the enhanced diffusivity causes microstructural changes within constituents and along the interfaces. The investigated CoNiAl-Ti composite proved to be consolable by SPS at 950 °C. Simultaneously, composite material underwent substantial structural changes.

On the titanium side, initial hcp A3-Ti phase (α-Ti) was affected by a long-range diffusion of Al, Ni and Co. Upon heating to sintering temperature (950 °C), titanium transforms from the hcp α-Ti phase to the bcc β-Ti phase and transforms back upon subsequent cooling. It is well known that the diffusivity of impurities in the β-Ti phase is by orders of magnitude higher than in α-Ti, namely the diffusion coefficient of Al in β-Ti is higher by more than three orders of magnitude just above the β-transus temperature^44^.

Aluminium atoms are soluble in Ti up to approx. 9 at.%, while at higher concentrations, Ti_3_Al particles form^45,46^. Ti_3_Al particles were not observed either by SEM or by TEM and therefore the Al content must have fallen below the 9 at.% limit, which is consistent with EDS results. More pronounced is the effect of Ni and Co. Enhanced diffusion during sintering (even for 1 min only) resulted in eutectic composition. In titanium rich Ti-Ni solution, the eutectic point is at around 5 at.% of Ni, while in Ti-Co solution it is approx. at 9 at.% of Co. The eutectic temperatures are 765 °C and 685 °C for Ni and Co, respectively; therefore well below the sintering temperature. Note that the total content of Co and Ni measured by area averaged EDS on titanium side was 7 wt.%, which perfectly corresponds to the eutectic composition of ternary Ti-Ni-Co solution. Upon cooling, a two phase eutectic structure consisting of hcp A3-Ti matrix and C9-Ti_2_(Co,Ni) particles is formed. The composition of particles could not be determined by EDS in SEM as they are small. However, the first interface layer from the Ti side has the very same structure as the small particles, i.e. C9-Ti_2_(Co,Ni), space group Fd-3m and the lattice parameter 11,3 Å. The overall content of Co and Ni in this layer may be determined by EBSD and was found to be in the range of 31–34 at. %, which corresponds well to the Ti_2_(Co,Ni) phase. As a consequence, the small particles may be assumed to belong to the same phase Ti_2_(Co,Ni), as well.

C9-Ti_2_(Co,Ni) interface layer adjoins the next interface layer with low aluminium content and composition satisfying rules for B2 phase: x_Co+Ni_ ≈ 0.5 and x_Ti_ ≈ 0.5. The diffraction experiments found a B2 phase with lattice parameter smaller than that of NiTi just on the detectability limit. The crystalline structure of this (Co,Ni)Ti layer (B2) was confirmed by SAED in TEM (cf. Fig. 8c).

Focusing on the CoNiAl side, we found that the matrix of the CoNiAl shape memory alloy phase has a B2 structure with the chemical composition very similar to the initial CoNiAl-SMA powder^36,37^. The interior of grains of the functional alloy is filled with the precipitates (white in contrast) whose composition is close to Co_2_NiAl. Nevertheless, all X-ray diffraction and neutron diffraction experiments revealed the A1 FCC cobalt structure with strong admixture of aluminium and nickel^37^. In fact, two morphologies of cobalt rich particles in B2-matrix can be distinguished in bulk CoNiAl samples: big, irregular particles with rounded contours and small rectangular particles. According to our previous investigations, we assume that rounded particles belong to the disordered A1 FCC cobalt phase, while small rectangular particles are ordered L1_2_ phase^38^. In our specimens the small rectangular particles in B2-(Co,Ni)Al matrix are also observed. However, the difference between ordered and disordered phase cannot be resolved by diffraction patterns, due to low volume fraction of the ordered phase and possibly low degree of ordering.

The main peculiarity of the investigated system is the similar role of titanium and aluminium in the alloying with Co and Ni. Thus, phases with the same or very similar crystallographic structure (in particular the two different B2 phases) are created on both sides of reacting interface.

Detailed SEM observation proved that there was another layer formed between the B2-(Co,Ni)Ti phase and B2-(Co,Ni)Al phase. This intermetallic phase contains significant and similar amount of all four elements. It could be therefore a mixed phase of (Co,Ni)(Al,Ti). Nevertheless, it is expected that the phase with such stoichiometry would be the L2_1_-ordered phase^40^. This phase, or better said, its real ordering was not resolved by both diffraction experiments and more detailed TEM investigation is needed to identify the details of ordering.

To conclude, interface layers were successfully identified by a series of advanced experimental methods. Starting from titanium side, phases across the Ti-CoNiAl interface were identified as: A3-Ti – A3-Ti + C9-Ti_2_(Co,Ni) – C9-Ti_2_(Co,Ni) – B2-(Co,Ni)Ti – L2_1_-(Co,Ni)(Al,Ti) – B2-(Co,Ni)Al – B2-(Co,Ni)Al + A1-(Co,Ni,Al).

Finally, note that the chemical composition of all formed phases is independent of CoNiAl:Ti ratio and more surprisingly also independent of the sintering time. The effect of sintering time on volume fraction of individual phases could not be resolved either by SEM or by diffraction experiments. Nevertheless, previous magnetization measurements^20^ showed a lower magnetization of samples sintered for longer time (5 min vs. 1 min), which suggests that the volume fraction of non-magnetic interface layers increased at the expense of magnetic CoNiAl alloy. Thus we can conclude that the short time sintering time is sufficient and favourable for the preparation of magnetic composite.

## Materials and Methods

**Figure 10:**
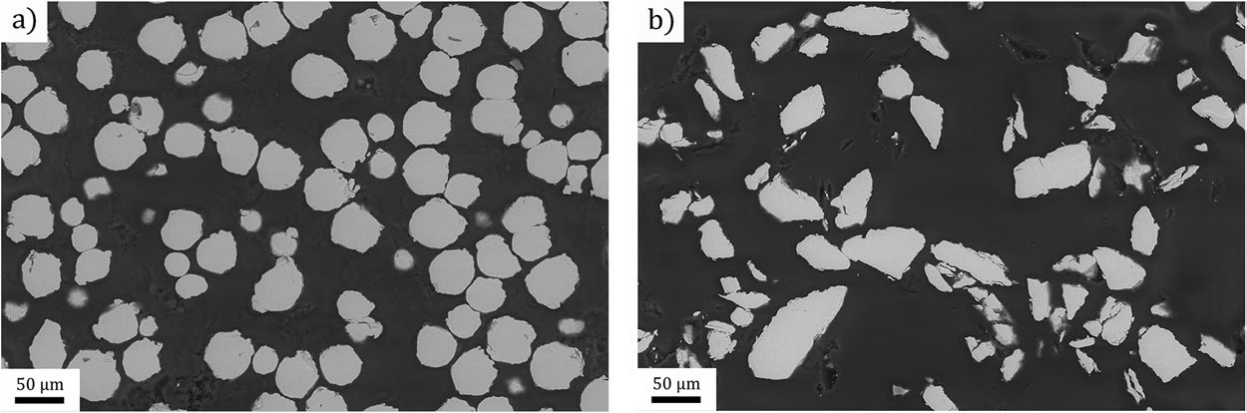
SEM micrographs of (**a**) CP-Ti and (**b**) Co_38_Ni_33_Al_29_ powders.

The Co_38_Ni_33_Al_29_ powder was prepared by mechanical grinding from a cast ingot having a rough dendritic microstructure and by subsequent milling in planetary mill. Resulting powder contained particles with irregular shapes and a diameter up to 100 µm (see Fig. 10). The powder was sieved to select the particles with the diameter between 20–63 µm. The composition of the powder obtained by EDS analysis was the following: X_Co_ = (39.9 ± 0.4) at.%; X_Ni_ = (33.8 ± 0.4) at.%; X_Al_ = (24.8 ± 0.3) at.% and iron contamination originating from grinding/milling X_Fe_ = (1.5 ± 0.1) at.%. The distribution of iron was not homogeneous.

Gas atomized CP-Ti (Grade 2) powder was purchased from TLS Technik GmbH & Co. Spezialpulver KG (Bitterfeld, Germany). The chemical composition as specified by the supplier revealed just a small contamination by Fe 0.07 at.% and an oxygen content of 0.42 at.% typical for Grade 2 commercial purity Ti. The powder was sieved by the supplier and the fraction of particle sizes in the diameter of 20–63 µm was used to have the particle size of both constituents approximately equal. More specifically, the nominal particle size distribution was the following: 22 wt.% particles below 35 μm, 38 wt.% 35–45 μm, 29 wt.% 45–55 μm, and 11 wt.% in the range of 55–65 μm. The powders were stored in air.

**Figure 11:**
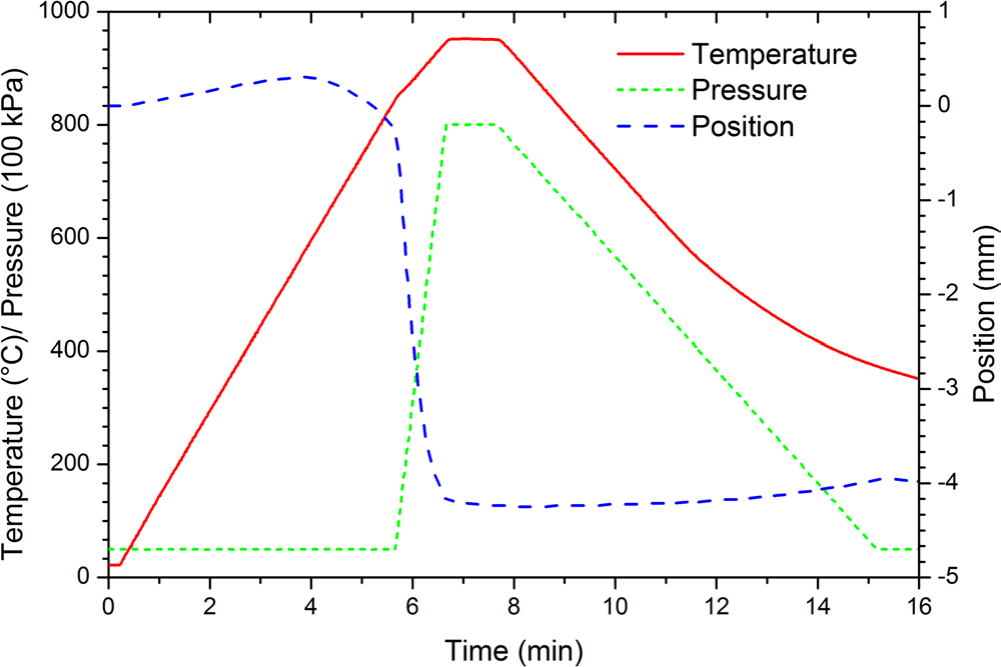
The time evolution of the SPS processing parameters for the sample prepared at 950 °C under the pressure 80 MPa for 1 min from the powders mixture CoNiAl:Ti in the ratio of 1:2. The 9 g of powder mixture was processed in a 20 mm wide die.

These two powders were mechanically mixed together for a few minutes in a closed container. Three different powder ratios were prepared, namely 2:1, 1:1, 1:2 of Co_38_Ni_33_Al_29_:Ti (hereafter referred to as CoNiAl:Ti). The mixed powder samples were sintered by spark plasma sintering device (10–4 SPS, Thermal Technologies LLC) at 950 °C and the pressure of 80 MPa for 1 min or 5 min, with the heating rate of 150 °C/min. An example of the sintering process parameters evolution is given in Fig. 11.

The cross-sections of powders and sintered samples were investigated. The specimens were ground and polished using diamond suspensions down to 1 μm. The final polishing was done using OP-S mixed with H_2_O_2_ and strongly diluted Kroll’s reagent (HNO_3_ and HF).

Scanning electron micrographs were taken employing scanning electron microscopes Zeiss Auriga Compact and Tescan FERA 3, both with EDS/EBSD analyser systems by EDAX. Transmission electron microscope (TEM) Jeol JEM 2200 equipped with STEM detector operated at an accelerating voltage of 200 kV was used for detail structure observations. The specimens (disks of the diameter of 3 mm) for TEM analysis were cut from the selected sample with the CoNiAl:Ti powder ratio of 2:1 sintered for 1 minute. Thin foil for TEM observations was obtained by electrochemical thinning using the double jet polisher Tenupol-5.

XRD measurements were carried out on the powder X-ray diffractometer D8 Discover in vertical Bragg-Brentano geometry (2.5° Soller slits in both primary and secondary beam and 0.5° divergence slit) with filtered Cu Kα radiation (Niβ filter was inserted in the secondary path). Measurement conditions were the following: the range from 10 to 120° in 2Θ, 0.08° 2Θ step size and 192 s counting time per step. Neutron powder diffraction measurements at room temperature were performed on diffractometer MEREDIT@NPI^47^ of CANAM infrastructure. Half-cylinder shape samples of the diameter of 18 mm and the thickness of 5 mm were placed in vanadium container and rotated along the vertical axis to minimize the influence of the potential sample texture. Diffraction patterns were collected between 4° and 144° of 2Θ with the step of 0.8° and using neutrons with a wavelength of 1.46 Å. Full pattern refinement was performed using a FullProf software^48^. In order to obtain higher reliability of phase determination the results of both diffraction methods were simultaneously used for pattern refinement.

Microstructural inhomogeneities in sintered samples were determined by SANS measurements at ambient temperature with thermal neutrons of the wavelength of 2.09 Å using the double-bent-crystal SANS instrument MAUD of CANAM infrastructure (NPL Řež)^49^. The double-crystal arrangement of the SANS experiment allows recording one dimensional data in the slit geometry. Three instrumental resolutions were combined for covering the scattering vector magnitude *Q* range from 2 × 10^−4^ to 1 × 10^−2^ Å^−1^. SASProfit program^50^ was used for data fitting.

The datasets generated during and/or analysed during the current study are available from the corresponding author on reasonable request.

## Conclusions

Spark plasma sintering method was successfully used to prepare dense composite of a light-weight metal (titanium) and shape memory alloy (Co_38_Ni_33_Al_29_) and its structure was investigated by a broad variety of microscopic and diffraction techniques.

The following conclusions can be drawn from this experimental study:

- Al, Co and Ni diffuse into Ti during sintering and eutectic two phase structure with C9-Ti_2_(Co,Ni) precipitates is formed.
- During sintering, CoNiAl decomposes into the B2-(Co,Ni)Al matrix and A1-(Co,Ni,Al) particles similarly as bulk CoNiAl during annealing.
- Complicated interface intermetallic structure containing C9-Ti_2_(Co,Ni), B2-(Co,Ni)Ti and L2_1_-(Co,Ni)(Al,Ti) was completely revealed.
- SANS measurement revealed that the volume fraction of small precipitates of C9-Ti_2_(Co,Ni) in A3-Ti matrix and A1-(Co,Ni,Al) in B2-(Co,Ni)Al matrix decreases with increasing sintering time, while the average size of the particles increases.
